# Developing a Hierarchical Model for the Spatial Analysis of PM_10_ Pollution Extremes in the Mexico City Metropolitan Area

**DOI:** 10.3390/ijerph14070734

**Published:** 2017-07-06

**Authors:** Alejandro Ivan Aguirre-Salado, Humberto Vaquera-Huerta, Carlos Arturo Aguirre-Salado, Silvia Reyes-Mora, Ana Delia Olvera-Cervantes, Guillermo Arturo Lancho-Romero, Carlos Soubervielle-Montalvo

**Affiliations:** 1 Department of Physics and Mathematics, Universidad Tecnológica de la Mixteca, 69000 Huajuapan de León, Oax., Mexico; sreyes@mixteco.utm.mx (S.R.-M.); ana.olvera@mixteco.utm.mx (A.D.O.-C.); lanchoga@mixteco.utm.mx (G.A.L.-R.); 2 Department of Statistics, Colegio de Postgraduados, Campus Montecillo, Texcoco, 56230 Montecillo, Mex., Mexico; hvaquera@colpos.mx; 3 Faculty of Engineering, Universidad Autónoma de San Luis Potosí, 78280 San Luis Potosí, S.L.P., Mexico; carlos.aguirre@uaslp.mx (C.A.A.-S.); carlos.soubervielle@uaslp.mx (C.S.-M.)

**Keywords:** air pollution, particulate matter, extreme value theory, Markov Chain Monte Carlo (MCMC), nonstationary

## Abstract

We implemented a spatial model for analysing ${\mathrm{PM}}_{10}$ maxima across the Mexico City metropolitan area during the period 1995–2016. We assumed that these maxima follow a non-identical generalized extreme value (GEV) distribution and modeled the trend by introducing multivariate smoothing spline functions into the probability GEV distribution. A flexible, three-stage hierarchical Bayesian approach was developed to analyse the distribution of the ${\mathrm{PM}}_{10}$ maxima in space and time. We evaluated the statistical model’s performance by using a simulation study. The results showed strong evidence of a positive correlation between the ${\mathrm{PM}}_{10}$ maxima and the longitude and latitude. The relationship between time and the ${\mathrm{PM}}_{10}$ maxima was negative, indicating a decreasing trend over time. Finally, a high risk of ${\mathrm{PM}}_{10}$ maxima presenting levels above 1000 μg/m${}^{3}$ (return period: 25 yr) was observed in the northwestern region of the study area.

## 1. Introduction

Air pollution in urban areas has become a major problem [1]. Increases in industry and urban traffic due to economic and population growth have led to an increase in gas and particulate emission that contribute to air pollution [2]. Among air pollutants, fine inhalable particles, known as particulate matter (PM) are associated with respiratory illnesses such as bronchitis, emphysema, asthma and other chronic obstructive pulmonary diseases [3]. PM can be classified by size; particles of 10 μm or less in diameter are called ${\mathrm{PM}}_{10}$. They can consist of diverse solid and liquid particles, which may contain chemical constituents such as nitrates, sulfates and organic carbon [4]. PM originates from factories, internal combustion engines, heating systems, volcanoes, and deserts, and can include dust particles formed through the mechanical breakdown of larger particles during agricultural and mining processes [5]. In the Mexico City metropolitan area, a study conducted by the U.S. Department of Energy (DOE) and Mexico’s Petróleos Mexicanos (PEMEX) showed that approximately 50% of the ${\mathrm{PM}}_{10}$ was composed of dust from roadways, construction, and bare land [6].

Previous studies on the damage caused to human health by breathable particulate matter have revealed an association between high concentrations of ${\mathrm{PM}}_{10}$ and mortality due to respiratory diseases [1,7]. To reduce exposure and minimize the adverse effects of these particulates on public health, several studies have been conducted with a focus on understanding the causes and factors related to the origin and flow of these particles [4,8]. Most of these studies have relied on continuous measurements of ${\mathrm{PM}}_{10}$ to predict future concentrations based on various models, such as multiple regression models, neural networks [9] and generalized linear models [5]. However, these short-term forecasting methodologies were developed for use in locations with limited infrastructure, and where obtaining continuous measurements of ${\mathrm{PM}}_{10}$ concentrations is difficult. In other, more densely populated regions, such as the Mexico City metropolitan area, there are systems that measure these concentrations and send information in real time to a central location for the immediate activation or deactivation of alerts or emergency procedures. In these cases, it is more convenient to study air pollutants through the use of robust methodologies such as the theory of extreme values.

Extreme value theory is used in many fields, such as environmental studies, engineering and finance, to monitor the quantitative risk of future extreme events [10,11]. In the environmental sciences, it is used to model extremes of temperature, rainfall, wind and pollution, among other phenomena. The asymptotic distribution of maxima is known as the generalized extreme value distribution, which is described by three parameters corresponding to location, scale and shape [12]. These parameters are estimated using the maximum likelihood method [13]. However, this method is not robust with a small sample size, so many other estimation methods have also been proposed, such as the method of moments, the use of L-moments and the use of weighted probability moments [14,15,16].

Recently, new methodologies have been proposed for the study of extreme values, mainly for application to hydroclimatological and environmental data, all of them based on the generalized extreme value distribution. For example: Gaetan and Grigoletto [17] proposed the use of Markov random fields approximated based on smoothing kernel parameters for modeling the parameters of the GEV distribution. Reich et al. [18] studied heat waves using a Bayesian hierarchical model with the generalized Pareto distribution (GPD). Cooley and Sain [19] studied maximum rainfall events by assigning a normal prior to the parameters of the GPD. Sang and Gelfand [11] studied the extreme values of spatial stochastic processes and modeled the observed trend as a function of covariates.

In a real scenario, it is common that conditions change and that the assumptions of stationarity that are required in a traditional analysis of extreme values not met; this is because there are often trends of extreme values [20,21,22]. Recent studies have introduced covariate functions for describing extreme value distributions to model either the location parameter, the scale parameter or both. Regarding the location parameter, Weissman [23] used a sine function, Tawn [24] and Scarf [25] proposed a linear function, Rosen and Cohen [26] and Pauli and Coles [27] used splines, and Bocci et al. [28] used a geoadditive model. In the case of the scale parameter, because this parameter is assumed to be positive, it is more common to model its logarithm; therefore, Yee and Stephenson [29] used additive models, El Adlouni et al. [10] and Rodriguez et al. [30] used linear functions, and Cannon [31] proposed the use of neural networks.

In this article, we present a spatio-temporal analysis of extreme ${\mathrm{PM}}_{10}$ values in the Mexico City metropolitan area. The objective of this study was to analyse ${\mathrm{PM}}_{10}$ data collected over time and in different spatial locations to gain insight into the distribution of ${\mathrm{PM}}_{10}$ maxima and to quantify the risk of future extreme ${\mathrm{PM}}_{10}$ pollution events, even in non-monitored regions.

## 2. Materials and Methods

### 2.1. Study Area

**Figure 1:**
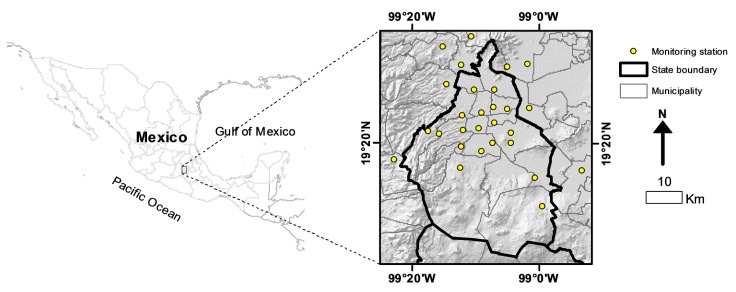
Study area.

The Mexico City metropolitan area is one of the world’s largest urban agglomerations, with approximately 25.4 million inhabitants spread over $7866\;{\mathrm{km}}^{2}$ at an average elevation of 2240 m above mean sea level. It is surrounded by mountains to the east and west, creating a basin with low points to the the north which leads to air pollution problems because of limited ventilation [6]. Two synoptic wind regimes prevail throughout the year: an anticyclonic westerly wind from November to April and a moist trade wind associated with the rainy season from May to October. Despite weak prevailing synoptic winds, the thermally induced local wind converges toward the city, which tends to restrict the ventilation of polluted air [32]. The study area and the locations of the primary sampling sites are shown in Figure 1.

### 2.2. Methodology

#### 2.2.1. A Nonstationary GEV Model

Let $Y_{1},\ldots ,Y_{n}$ be an independent and identically distributed set of random variables with distribution function $F_{X}\left(x\right)$ and let $M_{n}=max\left(Y_{1},\ldots ,Y_{n}\right)$. Let $F_{M_{n}}$ be the distribution function of $M_{n}$, because $F_{M_{n}}=F_{X}{\left(x\right)}^{n}$ we have that $M_{n}$ is a degenerate distribution when n→∞. The extreme value theory considers that the only nondegenerate limiting distribution $G_{n}=\left(M_{n}-a_{n}\right)/b_{n}$ (if such a sequences of constants $\left\{b_{n}>0\right\}$ and $\left\{a_{n}\right\}$ exist) as n→∞ is the generalized extreme value (GEV) distribution [12]:

$$F_{GEV}\left(y\right)=\left\{\begin{array}{cc} \exp\left\{-{\left(1+\kappa \frac{\left(y-\mu \right)}{\sigma }\right)}^{-\frac{1}{\kappa }}\right\}, & \kappa \neq 0,\\ \exp\left\{-\exp\left(-\frac{\left(y-\mu \right)}{\sigma }\right)\right\}, & \kappa =0 \end{array}\right.$$

for $y:1+\kappa \frac{\left(y-\mu \right)}{\sigma }>0$ when κ≠0, where −∞≤y≤μ+σ/κ when κ<0 (Weibull), −∞≤y≤+∞ when κ=0 (Gumbel) and μ+σ/κ≤y≤+∞ when κ>0 (Fréchet). Here, μ∈R, σ>0 and κ∈R are the location, scale and shape parameters, respectively; see [33].

In the nonstationary case, the parameters are expressed as a function of a vector of covariates $x_{t}$: $GEV\left(\mu \left(x_{t}\right),\sigma \left(x_{t}\right),\kappa \left(x_{t}\right)\right)$ [34]. To ensure a positive value for the scale parameter, $log\;\sigma (x_{t})$ is used instead of $\sigma (x_{t})$ in the estimation process.

#### 2.2.2. Proposed Approach

For the non-stationary case, consider assigning a linear predictor to the location and scale parameters. The κ parameter is usually assumed to be constant [29]. Therefore, we propose using the following conditions for the above parameters:

(1) $$\begin{array}{c} \mu_{t}=\sum_{i=1}^{P_{1}}X_{ti}\beta_{1i}+\sum_{i=1}^{P_{2}}Z_{ti}u_{1i},\\ log\;\sigma_{t}=\sum_{i=1}^{P_{1}}X_{ti}\beta_{2i}+\sum_{i=1}^{P_{2}}Z_{ti}u_{2i},\\ \kappa_{t}=\kappa \end{array}$$

where *X* is a scaled and centered $n\times p_{1}$ matrix of covariates that includes the intercept; $\beta_{i}$, i=1,2, is a vector of length $p_{1}$; $u_{i}$, i=1,2, is a vector of length $p_{2}$; *Z* is an $n\times p_{2}$ matrix such that ${\left\{Z\right\}}_{ij}=\exp\left[-{\left(∥{\underline{x}}_{i}-{\underline{k}}_{j}∥\right)}^{2}\right]$, i=1,…,n, $j=1,\ldots ,p_{2}$; ${\underline{x}}_{i}$ is the vector of covariates for the *i*-th observation, scaled and centered; and ${\underline{k}}_{j}$ the *j*-th centroid obtained using the method of average linkage hierarchical clustering [28,35].

#### 2.2.3. Maximum Likelihood Estimation

For a sample of n observations: $\underline{y}=\left(y_{1},\ldots ,y_{n}\right)$, the maximum likelihood estimator for the non-stationary GEV can be determined by maximizing the likelihood function

$$\begin{array}{c} L(\mu_{t},\sigma_{t},\kappa_{t}|\underline{y})=\prod_{t=1}^{n}\frac{1}{\sigma_{t}}\exp\left\{-{\left[1+\kappa_{t}\left(\frac{y_{t}-\mu_{t}}{\sigma_{t}}\right)\right]}^{-\frac{1}{\kappa_{t}}}\right\}\times {\left[1+\kappa_{t}\left(\frac{y_{t}-\mu_{t}}{\sigma_{t}}\right)\right]}^{-\left(1+\frac{1}{\kappa_{t}}\right)} \end{array}$$

where *n* is the number of observations. The function of $\kappa_{t}$ is usually assumed constant [29], so the log-likelihood is:

$$\begin{array}{c} \ell \left(\mu_{t},\sigma_{t},\kappa |\underline{y}\right)=-n\log\sigma_{t}-\sum_{t=1}^{n}{\left[1+\kappa \left(\frac{y_{t}-\mu_{t}}{\sigma_{t}}\right)\right]}^{-\frac{1}{\kappa }}-\sum_{t=1}^{n}\left(1+\frac{1}{\kappa }\right)\log\left[1+\kappa \left(\frac{y_{t}-\mu_{t}}{\sigma_{t}}\right)\right] \end{array}$$

Let $C=\left\lfloor X\;Z\right\rfloor$ and ${\underline{b}}_{\left(i\right)}^{\prime }=\left\lfloor {\underline{\beta }}_{\left(i\right)}^{\prime }\;{\underline{u}}_{\left(i\right)}^{\prime }\right\rfloor ,$ where *C* is a n×p matrix, with $p=p_{1}+p_{2};$${\underline{b}}_{\left(i\right)}^{\prime }$ is a vector of p×1 parameters, the linear predictors can be written as:

$$\mu_{t}=Cb_{1}\;;\;log\;\sigma_{t}=Cb_{2}\;;\;\kappa_{t}=\kappa$$

For this study, we assigned a penalization (P) to the vector of parameters, such that:

$$P=\left[\begin{array}{cc} \frac{1}{\sigma_{\beta }^{2}}⊗I_{p_{1}} & 0\\ 0 & \frac{1}{\sigma_{u}^{2}}⊗I_{p_{2}} \end{array}\right]$$

where $I_{p_{1}}$ and $I_{p_{2}}$ are identity matrices of order $p_{1}$ and $p_{1}$ respectively, $\sigma_{\beta }^{2}$ and $\sigma_{u}^{2}$ are values that control the degree of regularization of the model.

Therefore the penalized log-likelihood of the model is:

$$\ell_{n}^{p}\left(\mu_{t},\sigma_{t},\kappa |\underline{y}\right)=\sum_{t=1}^{n}\ell_{t}\left(\mu_{t},\sigma_{t},\kappa |\underline{y}\right)-\sum_{i=1}^{2}{\underline{b}}_{\left(i\right)}^{\prime }P{\underline{b}}_{\left(i\right)}-\frac{1}{\sigma_{\kappa }^{2}}\kappa^{2}$$

where:

$$\ell_{t}\left(\mu_{t},\sigma_{t},\kappa |\underline{y}\right)=-\log\sigma_{t}-{\left[1+\kappa \left(\frac{y_{t}-\mu_{t}}{\sigma_{t}}\right)\right]}^{\frac{1}{\kappa }}\log\left[1+\kappa \left(\frac{y_{t}-\mu_{t}}{\sigma_{t}}\right)\right]$$

Defining $\ell_{n}(\mu_{t},\sigma_{t},\kappa |\underline{y})=\sum_{t=1}^{n}\ell_{t}\left(\mu_{t},\sigma_{t},\kappa |\underline{y}\right)$ and $\ell_{PL}\left(b_{\left(1\right)},b_{\left(2\right)},\kappa \right)=\left[-\sum_{i=1}^{2}{\underline{b}}_{\left(i\right)}^{\prime }P{\underline{b}}_{\left(i\right)}-\frac{1}{\sigma_{\kappa }^{2}}\kappa \right]$ we can rewrite the log-likelihood as:

(2) $$\begin{array}{c} \ell_{n}^{p}\left(\mu_{t},\sigma_{t},\kappa |\underline{y}\right)=\ell_{n}\left(\mu_{t},\sigma_{t},\kappa |\underline{y}\right)+\ell_{PL}\left(b_{\left(1\right)},b_{\left(2\right)},\kappa \right) \end{array}$$

In order to compare two models, let $M_{1}$ with $\theta_{1}$ a parametric vector against another model $M_{0}$ with $\theta_{0}$ a subset vector such as $M_{1}\subset M_{0}$, a simple way to compare two models is to use the deviance statistic defined by [34]

$$D=2\left(l_{n}^{*}\left(M_{1}\right)-l_{n}^{*}\left(M_{0}\right)\right)$$

where $l_{n}^{*}\left(M\right)$ is the maximized log likelihood function of model M. Values of D greater than $\chi_{k}^{2}$ are considered significant, where *k* is the difference between the dimensions of $M_{1}$ and $M_{0}$, thus model $M_{1}$ explains better data variation than model $M_{0}$.

Penalized maximum likelihood estimators are used in this work for two reasons, the first is to use these estimators to perform a procedure of variables selection through the deviance, and second, we will use these estimators as initial values for the Bayesian hierarchical model to reduce the number of samples necessary to reach the stationary distribution of the MCMC algorithm.

#### 2.2.4. Bayesian Implementation

Under the assumption that $\pi \left(y_{t}|\theta_{t}\right)$ is the GEV distribution with parameters of $\theta_{t}=\left(\mu_{t},\sigma_{t},\kappa \right)$, a Bayesian formulation for the model of extreme values is as follows:

(3) $$\begin{array}{c} \pi \left(\theta_{t},\omega |y_{t}\right)\propto \pi \left(y_{t}|\theta_{t}\right)\pi \left(\theta_{t}|\omega \right)\pi \left(\omega \right) \end{array}$$

where $\omega =\left(\beta_{1},\beta_{2},u_{1},u_{2}\right)$ is such that the set of equations given in Equation (1) is satisfied. This hierarchical model consists of three levels: a data level, denoted by $\pi \left(y_{t}|\theta_{t}\right)$; a process level, denoted by $\pi \left(\theta_{t}|\omega \right)$; and a prior level, denoted by $\pi \left(\omega \right)$. Alternatively, the model given in Equation (3) with the conditions given in Equation (1) can be reformulated as a function of the parameter space of $\omega^{*}=\left(\beta_{1},\beta_{2},u_{1},u_{2},\kappa \right)$ and $\omega^{**}=\left\{\sigma_{1},\sigma_{2}\right\}$; therefore, we can write the posterior distribution as follows:

(4) $$\begin{array}{c} \pi \left(\omega^{*},\omega^{**}|y_{t}\right)\propto \pi \left(y_{t}|\omega^{*}\right)\pi \left(\omega^{*}|\omega^{**}\right)\pi \left(\omega^{**}\right) \end{array}$$

where $\pi \left(y_{t}|\omega^{*}\right)$ is the GEV density under the conditions on the parameters given in Equation (1). The prior distribution for $\omega^{*}$ is such that

$$\begin{array}{c} \beta_{1}∼N\left(0,10^{4}I\right)\\ \beta_{2}∼N\left(0,10^{4}I\right)\\ u_{1}|\sigma_{1}∼N\left(0,\sigma_{1}^{2}I_{K_{x}}\right)\\ u_{2}|\sigma_{2}∼N\left(0,\sigma_{2}^{2}I_{K_{x}}\right)\\ \kappa ∼Uniform(-5,5) \end{array}$$

The prior distribution for the hyperparameters $\omega^{**}$ is given by

$$\begin{array}{c} \sigma_{1}^{2}∼Half-Cauchy\left(25\right)\\ \sigma_{2}^{2}∼Half-Cauchy\left(25\right) \end{array}$$

To sample the a posteriori distribution, we use a MCMC method with an acceptance probability given by

$$\alpha \left(\theta^{*}|\theta \right)=min\left(1,\frac{\pi \left(x|\theta^{*}\right)\pi \left(\theta^{*}\right)Q\left(\theta^{*},\theta \right)}{\pi \left(x|\theta \right)\pi \left(\theta \right)Q\left(\theta ,\theta^{*}\right)}\right)$$

where $\pi \left(\theta \right)$ is the prior distribution for the parameters, $\pi \left(x|\theta \right)$ is the likelihood, and *Q* is the proposal function.

#### 2.2.5. Simulation Study

In this section, we examine the performance of the hierarchical GEV model defined in Equation (4) using simulated data. We simulated 500 extremes from a GEV model with two covariates, $x_{1}$ and $x_{2}$, corresponding to the longitude and latitude, respectively. The $x_{1}$ values were generated with equally spaced data in the range of 0 to 4π, and the values of the covariate $x_{2}$ were randomly selected from the interval $\left[0,4\right]$, with

(5) $$\begin{array}{c} \mu_{t}=sin\left(\frac{{\left(\sqrt{{\left(x_{1}-2\pi \right)}^{2}+{\left(x_{2}-2\pi \right)}^{2}}\right)}^{\frac{3}{2}}}{5}\right)\\ \sigma_{t}=\sigma =0.3\\ \kappa_{t}=\kappa =-0.1 \end{array}$$

For our model, we ran 70,000 iterations to obtain samples of the a *posteriori* density, after a burn-in period of 60,000 iterations, and applied thinning in every fifth iteration. The hierarchical model given in Equation (4) was fitted by setting $p_{2}=10$ in equation set Equation (1). The estimate of the shape parameter was −0.14. The true functions of μ and σ as functions of the covariates $x_{1}$ and $x_{2}$ are shown in Figure 1a,c, respectively. The function corresponding to μ that was chosen for the simulation is a function that cannot be separated based only on the main effects of the covariates, so it cannot be adjusted as in most traditional models for extreme values. The function for sigma is a flat function in the space covariate.

**Figure 2:**
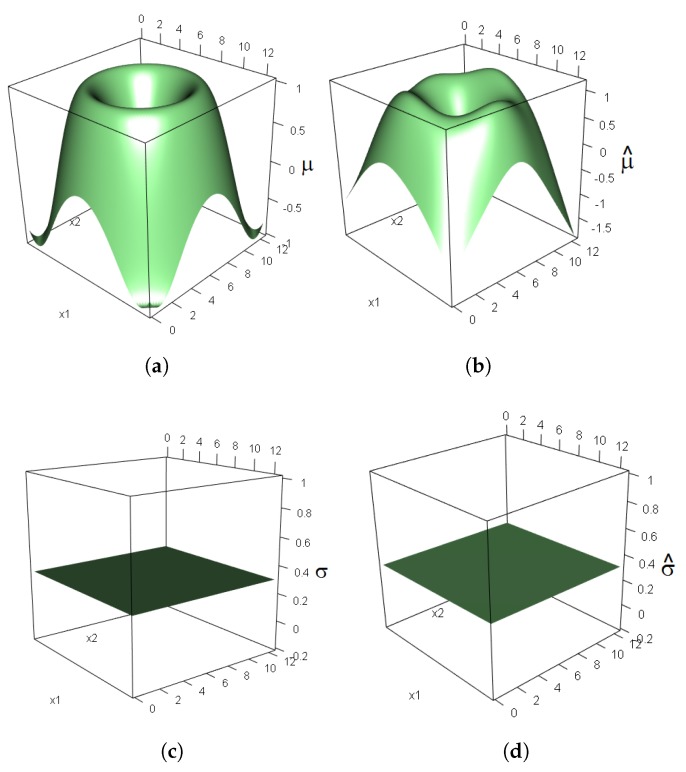
Real functions (**a**,**c**) and functions obtained by fitting the parameters (**b**,**d**) of a non-stationary generalized extreme value (GEV) model to simulated data with a sample size of n=500.

The true surface and local spatial patterns of the location parameter (Figure 2a) that were used to simulate the extreme values were recovered reasonably well by the smoothing function (Figure 2b). Similarly, a comparison of Figure 2c,d reveals that the smoothing function for the scale parameter of the extreme values based on the model given in Equation (1) recovered the true flat function for σ given in Equation (5).

To evaluate the performance of our model, we reserved a set of 1000 locations to serve as the testing set and then calculated the correlation with respect to their estimated values. Specifically, for the location parameter, we obtained a correlation of 0.99 between the predicted values and the testing data. Typically, when the trend found in the maxima can be modeled using nonlinear functions of a set of covariates, the interpretations of the individual coefficients of the smoothing function lose importance, and they are often meaningless. Therefore, the main information about the trend is provided by the smoothing function constructed from the complete set of estimated parameters.

#### 2.2.6. Maxima in ${\mathrm{PM}}_{10}$ Pollution Levels

##### Data Collection

The data correspond to 1238 observations of quarterly block maxima in ${\mathrm{PM}}_{10}$ between 1 January 1995, and 31 December 2016, obtained at 31 fixed monitoring stations of the Red Automática de Monitoreo Atmosférico (RAMA) network established by the Comision Ambiental Metropolitana of Mexico City to monitor compliance with ambient air quality standards; this network is part of the Sistema de Monitoreo Atmosférico (SIMAT), a program responsible for ongoing measurements of the main air pollutants in Mexico City.

##### Data Analysis

We constructed a GEV model of the ${\mathrm{PM}}_{10}$ maxima in the Mexico City metropolitan area, using multivariate smoothing functions of spatio-temporal covariates, latitude (s1), longitude (s2) and time (*t*), grouped into $X_{\left(1\right)}$ and meteorological covariates, wind speed (ws) and relative humidity (rh), grouped into $X_{\left(2\right)}$, to fit the trends in the non stationary GEV model. We defined two models to estimate the GEV parameters, the GEV0 model which included the spatio-temporal covariates and the GEV1 model that included the spatio-temporal covariates as well as the meteorological covariates.

According to the proposed approach in Equation (1), we define the joint contribution $\delta_{\theta_{t}}\left(X\right)$ of the set of covarites *X* corresponding to the $\theta_{t}$ parameter of the GEV distribution, as follows

(6) $$\begin{array}{c} \delta_{\mu_{t}}\left(X\right)=\sum_{i=1}^{p_{1}}X_{ti}\beta_{1i}+\sum_{i=1}^{p_{2}}Z_{ti}u_{1i},\\ \delta_{log\;\sigma_{t}}\left(X\right)=\sum_{i=1}^{p_{1}}X_{ti}\beta_{2i}+\sum_{i=1}^{p_{2}}Z_{ti}u_{2i},\\ \delta_{\kappa_{t}}\left(X\right)=\kappa \end{array}$$

The GEV0 model is a baseline model that incorporates the spatio-temporal covariates $X_{\left(1\right)}$ using a multivariate spline structure that implicitly includes the interaction between the covariables of the group, as follows

(7) $$\begin{array}{c} \mu_{t}=\delta_{\mu_{t}}\left(X_{\left(1\right)}\right),\\ log\;\sigma_{t}=\delta_{log\;\sigma_{t}}\left(X_{\left(1\right)}\right),\\ \kappa =\delta_{\kappa_{t}}\left(X_{\left(1\right)}\right) \end{array}$$

The GEV1 model is an extension of the GEV0 model that incorporates in an additive way the meteorological set of covariates $X_{\left(2\right)}$ with the structure given by Equation (6).

(8) $$\begin{array}{c} \mu_{t}=\delta_{\mu_{t}}\left(X_{\left(1\right)}\right)+\delta_{\mu_{t}}\left(X_{\left(2\right)}\right),\\ log\;\sigma_{t}=\delta_{log\;\sigma_{t}}\left(X_{\left(1\right)}\right)+\delta_{log\;\sigma_{t}}\left(X_{\left(2\right)}\right),\\ \kappa =\delta_{\kappa_{t}}\left(X_{\left(1\right)}\right)+\delta_{\kappa_{t}}\left(X_{\left(2\right)}\right) \end{array}$$

## 3. Results and Discussion

**Figure 3:**
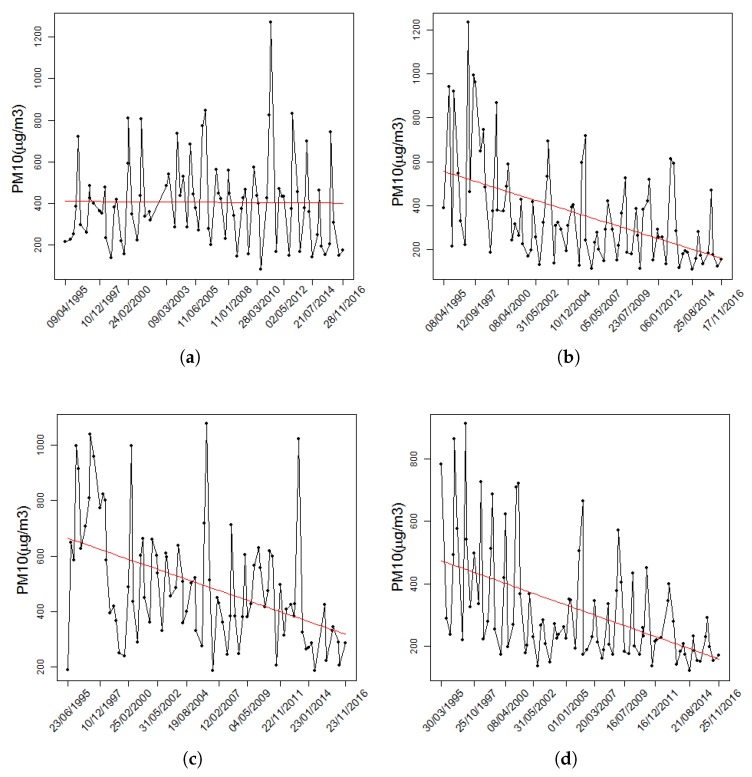
Example of quarterly block maxima of ${\mathrm{PM}}_{10}$ levels at (**a**) VIF = Villa Flores; (**b**) MER = Merced; (**c**) XAL = Xalostoc and (**d**) TLA = Tlanepantla. Linear trend over time are indicated in red.

A descriptive summary of the data is shown at Table 1. Four examples of quarterly block maxima of ${\mathrm{PM}}_{10}$ levels are presented in Figure 3. The U.S. and Mexican standard for ${\mathrm{PM}}_{10}$ pollution levels is 150 μg/m${}^{3}$. An analysis of Table 1 shows that in the area of Villa de las Flores, the permissible level was exceeded by more than three quarters of all measured extreme values. At three of the monitoring stations, CUA = Cuautitlán, NET = Netzahualcoyotl and XAL = Xalostoc, all observations exceeded the permitted standard level, this is because these locations have a high population density and more concentrated industry. The peak ${\mathrm{PM}}_{10}$ concentrations exceeded 1000 μg/m${}^{3}$ at CES = Cerro de la Estrella, MER = Merced, SAG = San Agustín, VIF = Villa de las Flores and XAL = Xalostoc; at SAG = San Agustín station, the level recorded was 10 times higher than the recommended safe limit.

**Figure 4:**
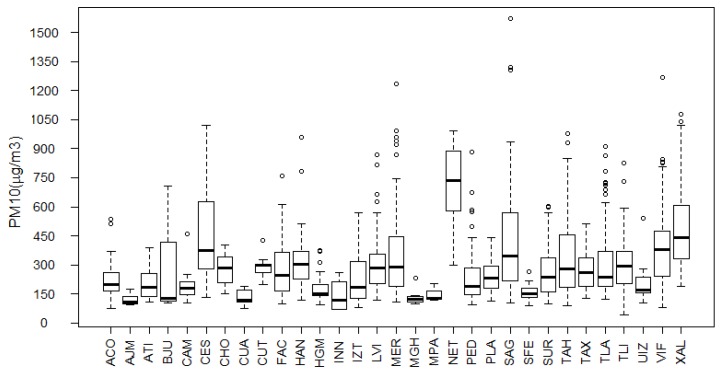
Boxplots of the ${\mathrm{PM}}_{10}$ maxima at 31 monitoring stations in the Mexico City metropolitan area.

Figure 4 shows boxplots of the ${\mathrm{PM}}_{10}$ maxima at each of the 31 monitoring stations considered in the study. The sites with lower ${\mathrm{PM}}_{10}$ maxima, less than 151 μg/m${}^{3}$ on average, are located in MGH = Miguel Hidalgo, MPA = Milpa Alta, AJM = Ajusco Medio and CUA = Cuajimalpa. Relatively well-preserved areas can still be found at these sites, which also have the lowest industrial activity and urban growth in the study area. By contrast, we also found sites with more than 723 μg/m${}^{3}$${\mathrm{PM}}_{10}$ on average, such as NET = Netzahualcoyotl, XAL = Xalostoc, CES = Cerro de la Estrella and MER = Merced, which are characterized by high industrial activity and heavily traveled paved and unpaved roads with heavy vehicular traffic [6].

In order to determine the significance of the meteorological variables in the non-stationary GEV model, we adjusted the GEV0 and GEV1 models by using the method of maximum likelihood (ML) and penalized maximum likelihood (PML) and we compared these two models through the deviance statistics. The results presented in Table 2 show that the model GEV1 is not statistically better than the baseline model GEV0, therefore the set of meteorological covariates wind speed (ws) and relative humidity (rh) did not present evidence at the 95% level to be included in the non-stationary model GEV for the modeling of ${\mathrm{PM}}_{10}$ maxima. Table 2 shows the effect of penalization of the parameters in the model, in the case of maximum likelihood method none regularization was performed, therefore $\ell_{PL}\left(b_{\left(1\right)},b_{\left(2\right)},\kappa \right)$ is considerably greater than the case of the penalized maximum likelihood. The maximum likelihood estimators have desirable statistical properties, however penalized estimators are preferred because they control the overfitting and reduce the instability of the estimates. In this work penalized estimators are used as initial values for the Bayesian hierarchical model, in order to reduce the number of chains necessary to reach the stationary distribution of the sampling MCMC algorithm.

Once the set of covariates involved in the model is determined, a hierarchical Bayesian model was fitted to analyse the ${\mathrm{PM}}_{10}$ maxima in the Mexico City metropolitan area. At the first level, we modeled the ${\mathrm{PM}}_{10}$ maxima using the generalized extreme value distribution; at the second level, we used multivariate smoothing spline functions to model nonstationary spatio-temporal extremes; and finally, at the third level, we assumed a priori distribution functions for the parameters of the model. Unfortunately, the posterior density in Equation (4) is not analytically tractable, so we implemented our model via the random walk Metropolis-Hastings algorithm to draw samples of the unknown parameters.

We performed the analysis using the statistical software *R* (version 3.3.1, R Foundation for Statistical Computing, Vienna, Austria). We fitted our Bayesian hierarchical model with the conditions expressed in Equation (6), setting $p_{2}=10$. We ran 70,000 MCMC samples after a burn-in period of 60,000 iterations, with thinning every fifth iteration. A look at the log-likelihood of the chain revealed convergence toward the stationary distribution of the MCMC algorithm.

Evidently, the extreme values of ${\mathrm{PM}}_{10}$ vary over time and from area to area because of local conditions, such as the topographic setting or the local wind. This fact justifies the modeling of the location parameter with respect to time and space. We verified this behavior by means of the estimates presented in Table 3, which show that the magnitudes of the studied maxima tend to decrease over time. Similarly, the boxplots in Figure 4 show that the distribution of the maxima is not the same in all monitored locations; therefore, a suitable model for the ${\mathrm{PM}}_{10}$ maxima must include temporal and spatial variations and their possible interactions. One of the strengths of our study is that the proposed model implicitly includes interactions between covariates, whereas most models consider only the main effects through additive or linear functions.

The estimates for the parameters of model Equation (1) corresponding to the coefficients of the main effects of the space-time covariates in the location and scale smoothing functions as well as the shape parameter are shown in Table 3. In this case, the vector of parameters $\beta_{1}$ corresponds to μ, and the vector $\beta_{2}$ corresponds to $log\;\sigma_{t}$. We noticed that the effect $\beta_{(1)t}$ of time on the location parameter is negative and that the effects $\beta_{(1)s1}$ and $\beta_{(1)s2}$ of the longitude and latitude are both positive. Because of the smoothing term in Equation (6) and the standardization of the covariates used in the model, it is not possible to establish a direct relation between the parameters estimated in Table 1 and the parameters of the GEV distribution in model Equation (6), except through the terms $\beta_{(1)0}$ and $\beta_{(2)0}$, which can be interpreted as the values of the location and scale parameters, respectively, of the GEV distribution in the middle of the studied period and in the center of the geographic area considered in the study.

**Figure 5:**
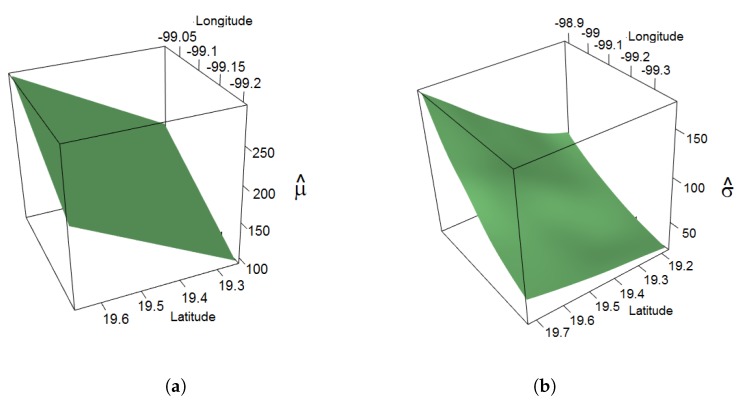
Estimated spatial smoothing of the (**a**) location parameter and (**b**) scale parameter in the year 2016.

The spatial smoothing for the location and scale functions is shown in Figure 5. The estimation function for the location parameter in the latitude–longitude coordinate system shows that the ${\mathrm{PM}}_{10}$ maxima tend to increase in the northwesterly direction. The scale parameter also increases in the northwesterly direction, but unlike for the location parameter, a slight decrease is observed in the region close to Iztacalco. The estimate of the shape parameter is 0.1715, and its 95% credible interval is (0.1694, 0.1737).

**Figure 6:**
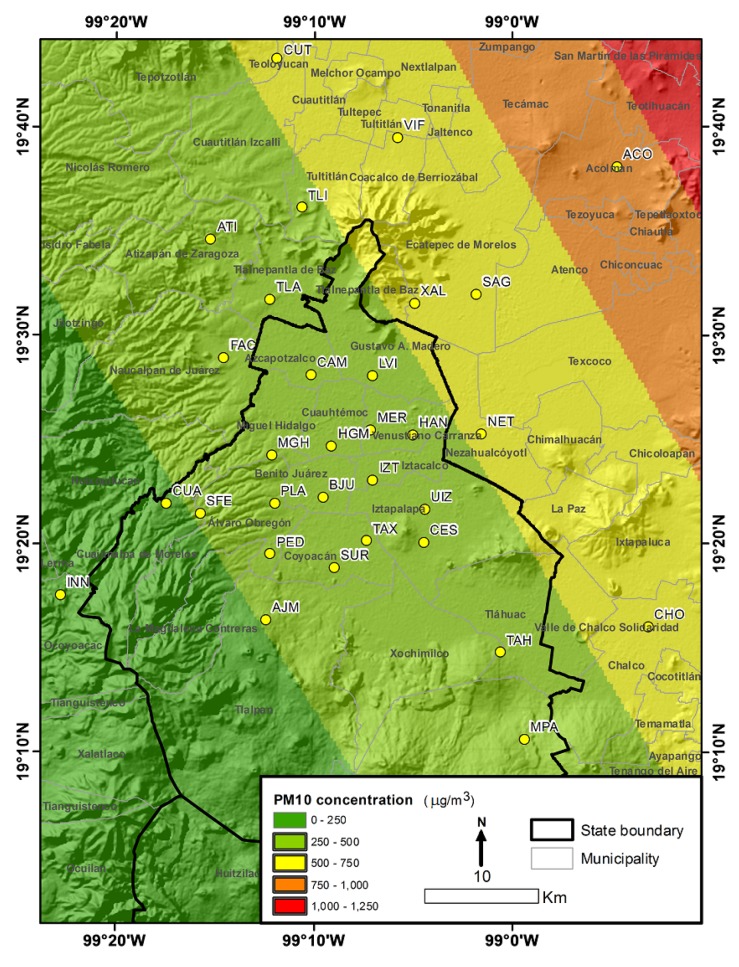
Return level surface for a return period of 25 years for the study region.

We have developed and validated a model of extreme values using a robust and solidly-based theory, an important application of the results of our study is through a risk map or return level map. The return level $Z_{p}$ is the threshold at which an extreme value is exceeded with probability p, which is expected to occur once every 1/p years [34]. Figure 6 shows the return levels for a return period of 25 years. Accordingly, the trend of increased risk in the northwest of the study area is preserved, with the greatest levels of risk in areas close to VIF = Villa Flores, SAG = San Agustín and ACO = Acolman and a lower level of risk in the area surrounding Pedregal.

Previous studies have analysed extremes of air pollutants using the generalized extreme value distribution [30,36]. However, the majority of these investigations have used information from a single site and consequently have ignored the aspect of spatial variability, which may result in underestimation of the GEV parameters. Several approaches to the spatio-temporal modeling of extreme values [19,28] have been implemented for environmental extreme data analysis. Therefore, on the basis of these studies, we have proposed a model that offers structural advantages for the modeling of extreme values.

Similar to the findings reported by studies in other countries [3], the extreme ${\mathrm{PM}}_{10}$ concentrations studied here exhibited spatial variations within the study area.

These results were consistent with the environmental characteristics of each monitored region: the locations with the highest industrial and population densities showed higher concentrations of ${\mathrm{PM}}_{10}$, and consequently, in these locations, the GEV model yielded high estimates of the location and scale parameters and the 25-year return map showed greater risks of extreme future events.

Most ${\mathrm{PM}}_{10}$ modeling studies on short-term dynamics have found that weather covariables such as wind speed or temperature are significant in the model [37]. This is not always the case in studies with a longer time horizon, where the conclusions for the long term are more robust. One of the reasons is due to the temporality of the data, daily maxima present a different association with respect to the meteorological covariates than the maxima obtained in a longer time window, such as quarterly maxima.

One of the findings of our study is the negative contribution of the effect of time on the trend of ${\mathrm{PM}}_{10}$ maxima; an explanation of this phenomenon is the continuous implementation of new emission control policies and the continuous revision of environmental norms in the Mexico City, which have recently become increasingly strict, mainly by regulating the traffic and restricting the automotive vehicles that can be used i.e., vehicle’s model year. Additionally, the Mexico City government have implemented a website for monitoring air quality (http://www.aire.cdmx.gob.mx/default.php), thus environmental contingency alerts are activated to mitigate the adverse effect of air pollutants on public health.

## 4. Conclusions

Recent years have seen a growing interest in the monitoring of ${\mathrm{PM}}_{10}$ air pollution because of the multiple health problems it causes in densely populated areas. In the Mexico City metropolitan area, ${\mathrm{PM}}_{10}$ air pollution often originates from dust from roadways and organic black carbon formed during combustion processes. This has led to is an ongoing public health problem over the past several years affecting a large section of the population. Recently, due to population growth and the increase in the number of combustion vehicles and industries, the ${\mathrm{PM}}_{10}$ air pollution problem has worsened. Therefore, it is extremely important to study the spatio-temporal trend of the ${\mathrm{PM}}_{10}$ maxima and provide this information to the management authorities so that prevention standards and policies can be reviewed and adapted to prevent extreme cases of ${\mathrm{PM}}_{10}$ air pollution.

Because wind speed (ws) and relative humidity (rh) are statistically significant covariates in most models for the short-term forecast for ${\mathrm{PM}}_{10}$ concentration, we consider including these covariates in the non-stationary GEV model. However by using the deviance statistic and the chi-square test, we found that at a credible level of 95%, the model with these covariates was not statistically better than the model that did not include these variables. Therefore, wind speed and relative humidity were not statistically significant in the non-stationary GEV model with quarterly block maxima of ${\mathrm{PM}}_{10}$ levels. The return levels for a return period of 25 years revealed a clear spatial trend of increased levels of ${\mathrm{PM}}_{10}$ in the northwest study area and a decreasing trend in the extreme values over time.

In this study, we implemented a methodology to analyse the nonstationary extreme data and to perform a spatio-temporal study of the maxima of breathable particulate matter pollution (${\mathrm{PM}}_{10}$) levels in the Mexico City metropolitan area. These ${\mathrm{PM}}_{10}$ levels usually vary in space and time, and can potentially include significant spatio-temporal interactions. Therefore, the commonly used models can underperform the GEV estimates and consequently result in inaccuracies. We achieved this using an analysis of non-stationary extreme values, in which we used a combination of existing traditional statistical techniques. We used the maximum likelihood method to perform a variable selection step, the penalized maximum likelihood estimators to obtain initial values and reduce the convergence time of the MCMC algorithm and fitted the estimators using a Bayesian approach to eliminate potential invalid parameter values. The combination of these statistical techniques gives support and solidity to our results.

We proposed a modification to the Bayesian estimation methods used in the previous studies related to the analysis of extreme values applied to environmental areas. In our model, the covariates are included in the generalized extreme value distribution through multivariate spline functions and, therefore, interactions between the covariates are also considered in the model. We observed that this approach can be easily extended to the modeling of extreme events and the generation of risk maps for air pollution, rainfall, heat waves, wind speed, etc. The results of the simulations conducted led us to conclude that the methodology is adequate and reliable for this type of study. Additionally, as a first attempt, time has been treated as a covariate. Extensions of this work should consider a more general model for spatio-temporal analysis, a specific analysis of the prior distributions of the parameters, and a method for determining the correct number of nodes in the multivariate spline functions.

## Figures and Tables

**Table 1:** Descriptive summary information on the extreme values of particulate matter less than 10 micrograms in diameter (${\mathrm{PM}}_{10}$) at the stations considered in the study.

Name	Symbol	Long$\left(W\right)$	Lat$\left(N\right)$	Min.	1st Qu.	Median	Mean	3rd Qu.	Max.
Acolman	ACO	99${}^{{}^{\circ}}$07${}^{\prime }$ 03.89${}^{\prime \prime }$	19${}^{{}^{\circ}}$28${}^{\prime }$04.4${}^{\prime \prime }$	76	166.2	197.5	230.5	252.5	535
Ajusco Medio	AJM	99${}^{{}^{\circ}}$12${}^{\prime }$16.54${}^{\prime \prime }$	19${}^{{}^{\circ}}$31${}^{\prime }$44.67${}^{\prime \prime }$	92	99	109	121.1	137	175
Atizapan	ATI	99${}^{{}^{\circ}}$04${}^{\prime }$56.64${}^{\prime \prime }$	19${}^{{}^{\circ}}$31${}^{\prime }$33.58${}^{\prime \prime }$	108	137	185	203.9	256	387
Benito Juárez	BJU	99${}^{{}^{\circ}}$07${}^{\prime }$10.53${}^{\prime \prime }$	19${}^{{}^{\circ}}$25${}^{\prime }$28.59${}^{\prime \prime }$	102	118.5	127	265.8	274.2	707
Camarones	CAM	99${}^{{}^{\circ}}$12${}^{\prime }$14.88${}^{\prime \prime }$	19${}^{{}^{\circ}}$19${}^{\prime }$30.52${}^{\prime \prime }$	102	150	181.5	189.8	213.2	462
Cerro de la Estrella	CES	99${}^{{}^{\circ}}$04${}^{\prime }$28.84${}^{\prime \prime }$	19${}^{{}^{\circ}}$20${}^{\prime }$05.03${}^{\prime \prime }$	130	279	373	444	617.5	1023
Chalco	CHO	99${}^{{}^{\circ}}$01${}^{\prime }$34.02${}^{\prime \prime }$	19${}^{{}^{\circ}}$25${}^{\prime }$16.14${}^{\prime \prime }$	150	207.5	283	272.3	341	401
Cuajimalpa	CUA	99${}^{{}^{\circ}}$10${}^{\prime }$37.82${}^{\prime \prime }$	19${}^{{}^{\circ}}$36${}^{\prime }$09.15${}^{\prime \prime }$	75	108	119	131.7	168	191
Cuautitlán	CUT	99${}^{{}^{\circ}}$05${}^{\prime }$47.72${}^{\prime \prime }$	19${}^{{}^{\circ}}$39${}^{\prime }$29.60${}^{\prime \prime }$	198	267.2	297	289.5	305	427
FES Acatlán	FAC	99${}^{{}^{\circ}}$00${}^{\prime }$38.03${}^{\prime \prime }$	19${}^{{}^{\circ}}$14${}^{\prime }$47.25${}^{\prime \prime }$	98	167.5	248	272.3	364	758
Hangares	HAN	99${}^{{}^{\circ}}$08${}^{\prime }$59.97${}^{\prime \prime }$	19${}^{{}^{\circ}}$18${}^{\prime }$52.12${}^{\prime \prime }$	117	228	302	333.8	369	959
Hospital General de México	HGM	99${}^{{}^{\circ}}$14${}^{\prime }$36.68${}^{\prime \prime }$	19${}^{{}^{\circ}}$28${}^{\prime }$56.90${}^{\prime \prime }$	96	143.8	153	190	193.2	376
Investigaciones Nucleares	INN	99${}^{{}^{\circ}}$05${}^{\prime }$01.04${}^{\prime \prime }$	19${}^{{}^{\circ}}$25${}^{\prime }$13.86${}^{\prime \prime }$	69	71.25	120	142	190.8	259
Iztacalco	IZT	99${}^{{}^{\circ}}$12${}^{\prime }$00.39${}^{\prime \prime }$	19${}^{{}^{\circ}}$21${}^{\prime }$57.12${}^{\prime \prime }$	78	128.5	186	230.8	317	569
La Villa	LVI	99${}^{{}^{\circ}}$01${}^{\prime }$49.16${}^{\prime \prime }$	19${}^{{}^{\circ}}$31${}^{\prime }$58.68${}^{\prime \prime }$	118	203.8	286	309	355.5	871
Merced	MER	99${}^{{}^{\circ}}$07${}^{\prime }$23.53${}^{\prime \prime }$	19${}^{{}^{\circ}}$20${}^{\prime }$08.48${}^{\prime \prime }$	109	187.5	290.5	357.8	437	1233
Miguel Hidalgo	MGH	99${}^{{}^{\circ}}$07${}^{\prime }$03.50${}^{\prime \prime }$	19${}^{{}^{\circ}}$23${}^{\prime }$03.88${}^{\prime \prime }$	100	109	121	134.7	137	230
Milpa Alta	MPA	98${}^{{}^{\circ}}$54${}^{\prime }$43.21${}^{\prime \prime }$	19${}^{{}^{\circ}}$38${}^{\prime }$07.8${}^{\prime \prime }$	119	123.5	128	150.3	166	204
Netzahualcoyotl	NET	99${}^{{}^{\circ}}$10${}^{\prime }$11.25${}^{\prime \prime }$	19${}^{{}^{\circ}}$28${}^{\prime }$06.25	298	580.5	737	722.4	887	991
Pedregal	PED	99${}^{{}^{\circ}}$04${}^{\prime }$25.96${}^{\prime \prime }$	19${}^{{}^{\circ}}$21${}^{\prime }$38.85	94	146	189	233	284	884
Plateros	PLA	99${}^{{}^{\circ}}$09${}^{\prime }$07.94${}^{\prime \prime }$	19${}^{{}^{\circ}}$24${}^{\prime }$41.82	112	178	233	241.2	294	440
San Agustín	SAG	99${}^{{}^{\circ}}$15${}^{\prime }$46.31${}^{\prime \prime }$	19${}^{{}^{\circ}}$21${}^{\prime }$26.48${}^{\prime \prime }$	104	216.5	346	430.9	571	1570
Santa Fe	SFE	99${}^{{}^{\circ}}$11${}^{\prime }$54.96${}^{\prime \prime }$	19${}^{{}^{\circ}}$43${}^{\prime }$19.86${}^{\prime \prime }$	91	131	149	159.2	182	267
Santa Ursula	SUR	98${}^{{}^{\circ}}$53${}^{\prime }$09.91${}^{\prime \prime }$	19${}^{{}^{\circ}}$16${}^{\prime }$01.01${}^{\prime \prime }$	100	164	237	265.8	335	603
Tlahuac	TAH	99${}^{{}^{\circ}}$15${}^{\prime }$14.87${}^{\prime \prime }$	19${}^{{}^{\circ}}$34${}^{\prime }$37.06${}^{\prime \prime }$	91	183	281	336.9	463.2	977
Taxqueña	TAX	99${}^{{}^{\circ}}$17${}^{\prime }$30.13${}^{\prime \prime }$	19${}^{{}^{\circ}}$21${}^{\prime }$55.12${}^{\prime \prime }$	128	188.5	262	280.6	334.5	513
Tlalnepantla	TLA	99${}^{{}^{\circ}}$12${}^{\prime }$09.57${}^{\prime \prime }$	19${}^{{}^{\circ}}$24${}^{\prime }$14.58${}^{\prime \prime }$	121	190	236	317.3	374	912
Tultitlán	TLI	99${}^{{}^{\circ}}$12${}^{\prime }$27.87${}^{\prime \prime }$	19${}^{{}^{\circ}}$16${}^{\prime }$19.77${}^{\prime \prime }$	41	203.2	293	303	368.5	828
UAM Iztapalapa	UIZ	99${}^{{}^{\circ}}$09${}^{\prime }$34.54${}^{\prime \prime }$	19${}^{{}^{\circ}}$22${}^{\prime }$13.67${}^{\prime \prime }$	105	158	172	201.8	238	539
Villa de las Flores	VIF	99${}^{{}^{\circ}}$22${}^{\prime }$49.87${}^{\prime \prime }$	19${}^{{}^{\circ}}$17${}^{\prime }$31.08${}^{\prime \prime }$	82	243.8	380.5	405.8	470.8	1269
Xalostoc	XAL	98${}^{{}^{\circ}}$59${}^{\prime }$24.68${}^{\prime \prime }$	19${}^{{}^{\circ}}$10${}^{\prime }$36.83${}^{\prime \prime }$	187	330	443	497	609	1076

**Table 2:** Statistical comparison of the GEV0 model against the GEV1 model.

% Method	Model	$\ell_{n}\left(\underline{y}|\mu_{t},\sigma_{t},\kappa \right)$	$\ell_{\mathrm{PL}}\left(b_{\left(1\right)},b_{\left(2\right)},\kappa \right)$	Deviance	*p*-value
ML	GEV0	−4607.8	−21,763.3	32	0.12
	GEV1	−4591.8	−21,737.2		
Penalized ML	GEV0	−4845.8	−28.1	26.6	0.33
	GEV1	−4832.5	−28.7		

**Table 3:** Estimates and 95% credible intervals of the nonstationary GEV model for the ${\mathrm{PM}}_{10}$ maxima.

% Parameter	Mean	95% CI
$\beta_{(1)0}$	235.6905	(235.367,236.0144)
$\beta_{(1)t}$	−46.1495	(−46.4354,−45.8724)
$\beta_{(1)s1}$	23.609	(23.3706,23.8377)
$\beta_{(1)s2}$	25.6023	(25.3444,25.8610)
$\beta_{(2)0}$	4.6134	(4.6112,4.6157)
$\beta_{(2)t}$	−0.3365	(−0.3387,−0.3344)
$\beta_{(2)s1}$	0.2200	(0.2179,0.2220)
$\beta_{(2)s2}$	0.1687	(0.1667,0.1707)
κ	0.1715	(0.1694,0.1737)
